# The complete chloroplast genome sequence of *Rosa* ‘Limoncello’ (Rosales: Rosaceae)

**DOI:** 10.1080/23802359.2023.2290854

**Published:** 2023-12-18

**Authors:** Jing Wang, Qiu-lan Xie, En-you Feng, Xiao-fei Liu

**Affiliations:** a Zhanjiang University of Science and Technology, Zhanjiang, China; b Zhanjiang Academy of Agricultural Sciences, Zhanjiang, China; c Environmental Horticulture Institute, Guangdong Academy of Agricultural Sciences, Guangdong Key Laboratory of Ornamental Plant Germplasm Innovation and Utilization, Key Laboratory of Urban Agriculture in South China, Ministry of Agriculture, Guangzhou, China

**Keywords:** Complete chloroplast genome, phylogenetic analysis, *Rosa* ‘limoncello’

## Abstract

*Rosa* ‘Limoncello’ finds applications in gardening and landscaping. In this study, we assembled and annotated the complete chloroplast genome of this variety for the first time. The length of its chloroplast genome was 156,493 bp, containing two short inverted repeat regions of 26,052 bp, each separated by a large single-copy region of 85,649 bp and a small single-copy region of 18,740 bp. The chloroplast DNA of *R.* ‘Limoncello’ consisted of 135 genes, including 90 protein-coding genes, eight ribosomal RNA genes, and 37 transfer RNA genes. On comparing the complete chloroplast sequence of *R.* ‘Limoncello’ with that of other *Rosa* species, *R.* ‘Limoncello’ was found to be closely related to *Rosa cymosa*. Thus, information on the chloroplast genome sequence of this rose variety can facilitate phylogenetic studies of the genus *Rosa*.

## Introduction

**Figure 1.**
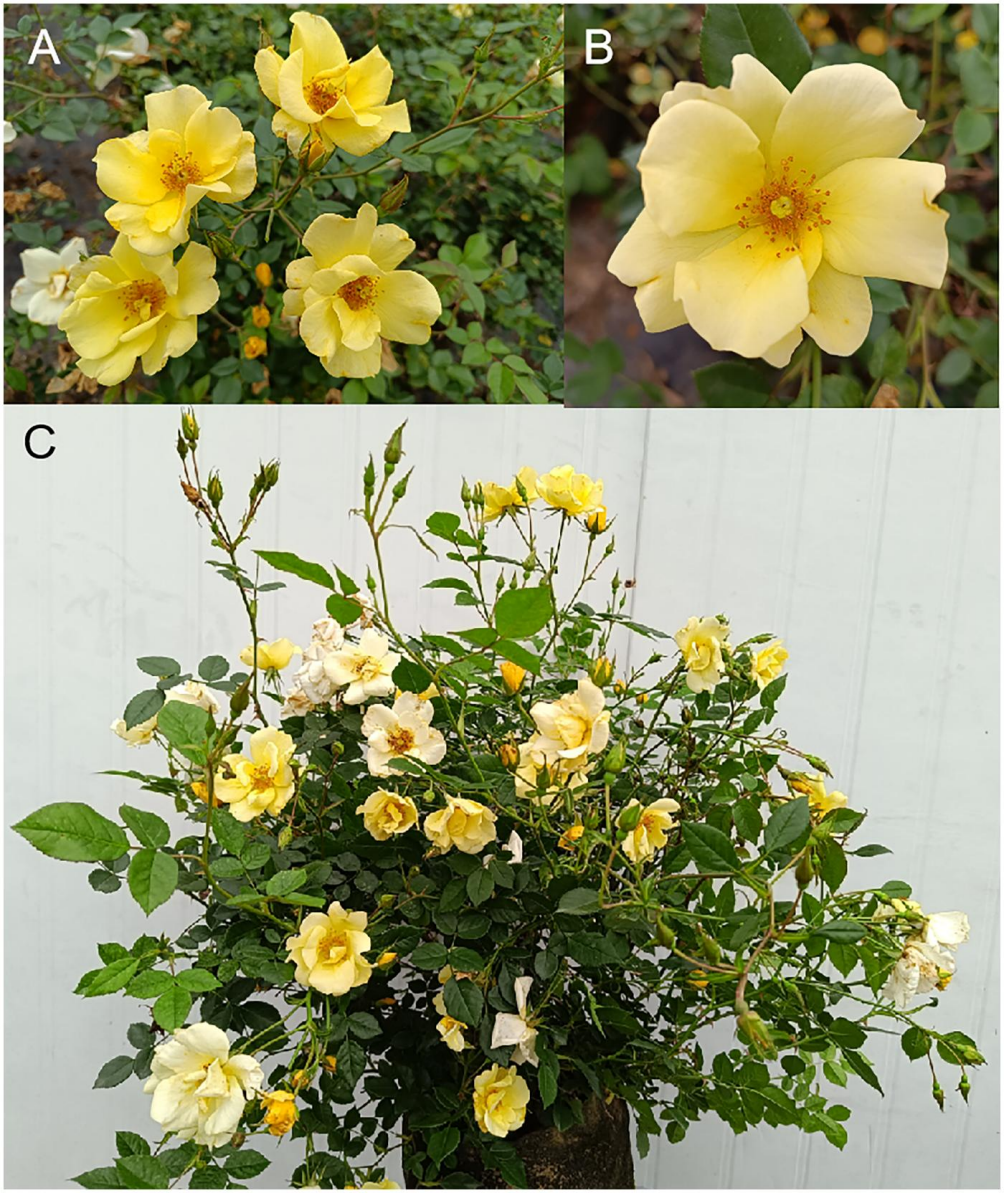
Morphology of *Rosa* ‘Limoncello’. (A) Flowering branch; (B) flower; (C) individual of *R.* ‘Limoncello’. The photos were taken by Qu Du at the Garden practice base of Zhanjiang University of Science and Technology, Zhanjiang, Guangdong, China (21°17′N, 110°25′E), in April 2023, without any copyright issues.

*Rosa* ‘Limoncello’ is a floribunda rose plant of the genus *Rosa* (Rosaceae) (Figure 1). It is a hybrid bred by Meilland International (Le Cannet-des-Maures, France) in 2008, and introduced in the United States by Star Roses & Plants (Sultana, CA) in 2009 as ‘Limoncello’ (Alain A 2010). *R.* ‘Limoncello’ is a shrub, 60–105 cm in height, with glabrous and glossy dark green leaves arranged on both sides of the stem. Its flowers are lemon-yellow, but they gradually turn light yellow with age. Stamens are golden. The corolla is yellow, with 5–7 individual petals. It is highly resistant blackspot and powdery mildew and has the characteristics of low maintenance and easy cultivation. *R.* ‘Limoncello’ is widely used in flower beds and garden borders or as potted plants.

Rosaceae is one of the most diverse angiosperm families with 124 genera and 3300 species with a global distribution. Rose genus plants have high ornamental value. Due to the diversity of genetic information, it is an essential and precious material to carry out Rose hybrid breeding practice and theoretical research. Many researchers have therefore carried out hybrid breeding work of the Rose genus and selected many varieties for use as cut flowers, potted plants, and garden ornamentals. Because of its simple structure, vital conservation, and amenability to sequencing, the chloroplast genome has been used in genetic structural and phylogenetic analyses of a wide range of plant taxa. The chloroplast genomes of many various species of Rose genus have also been published. However, no research reports have appeared on *R.* ‘Limoncello’ chloroplast genomes. Thus, information on the chloroplast genome sequence of this new rose variety can facilitate phylogenetic studies of the genus *Rosa*.

## Materials

The *R.* ‘Limoncello’ sample in this study was planted in Zhanjiang, Guangdong, China (21°17′N, 110°25′E). The chloroplast genome DNA of *R.* ‘Limoncello’ was extracted from fresh leaves. The specimen and extracted DNA have been deposited at the herbarium of Zhanjiang University of Science and Technology (contact person: Qu Du; e-mail: 1370592206@qq.com) under voucher number ZJUSTYLJDLC002.

## Methods

In this study, the genome DNA of *R.* ‘Limoncello’ was extracted from fresh leaves using a plant genomic DNA extraction kit (Sangon Biotech Co., Ltd., Shanghai, China). Extracted DNA was sheared into 300-bp fragments using the Covaris S220 ultrasonicator (Covaris, Woburn, MA). After mechanical (ultrasound) fragmentation, the extracted DNA was subjected to purification, end repair, poly(A) tail addition, and ligation of sequencing adapters and then analyzed by agarose gel electrophoresis for fragment size selection. PCR amplification was performed to generate a sequencing library, and the qualified library was sequenced on the Illumina NovaSeq platform (San Diego, CA). A paired-end library was constructed using the NEBNext^®^ Ultra™ DNA Library Prep Kit (New England Biolabs, Ipswich, MA). Whole chloroplast genome sequencing was performed using the Illumina platform (Illumina, San Diego, CA) at Sangon Biotech Co., Ltd. (Shanghai, China). Paired-end Illumina raw reads were filtered using Trimmomatic (Bolger et al. 2014), which were mapped to the chloroplast genome of the reference species (GenBank accession number: NC_051550). Bowtie 2 v2.2.4 (Langmead and Salzberg 2012) was used to exclude reads of nuclear and mitochondrial origin. SPAdes v3.10.1 (Bankevich et al. 2012) was used for *de novo* assembly of chloroplast genomes. Gaps in the obtained contigs were removed by splicing using GapFiller v1.11 (Boetzer and Pirovano 2012). PrInSeS-G v1.0.0 (Massouras et al. 2010) corrected base errors and indels in small fragments during splicing. The genomes were annotated using CPGAVAS2 (Shi et al. 2019), and a circular representation was generated with OGDRAW (Liu et al. 2023).

A phylogenetic tree was constructed by the maximum-likelihood (ML) method using entire chloroplast genomes. The ML analysis was performed using RAxML-HPC BlackBox v.8.1.24 at the CIPRES Science Gateway website based on the best-fit model of evolution (GTR + G) with 1000 bootstrap replicates. The GenBank accession numbers of the analyzed plant genomes are as follows: *Rosa xanthina* (NC_051543), *R. praelucens* (NC_037492), *R. banksiae* (NC_042194), *R. roxburghii* (NC_032038), *R. laevigata* (NC_046824), *R. laevigata* var. *leiocarpa* (NC_047418), *R. canina* (NC_047295), *R. filipes* voucher *ZZM1250-1* (NC_053856), *R. chinensis* var. *Spontanea* (NC_038102), *R. sterilis* (nom.nud.) (NC_053909), *R. cymosa* (NC_051550), *R. multiflora* (NC_039989), *R. maximowicziana* (NC_040960), *R. lucieae* (NC_040997), *Malus pumila* (MW115599), *Camellia rhytidophylla* (MT663343), *Hippeastrum reticulatum* (MT701523), and *Spathiphyllum cannifolium* (MK372232). The latter two species were used as outgroups.

## Results

**Figure 2.**
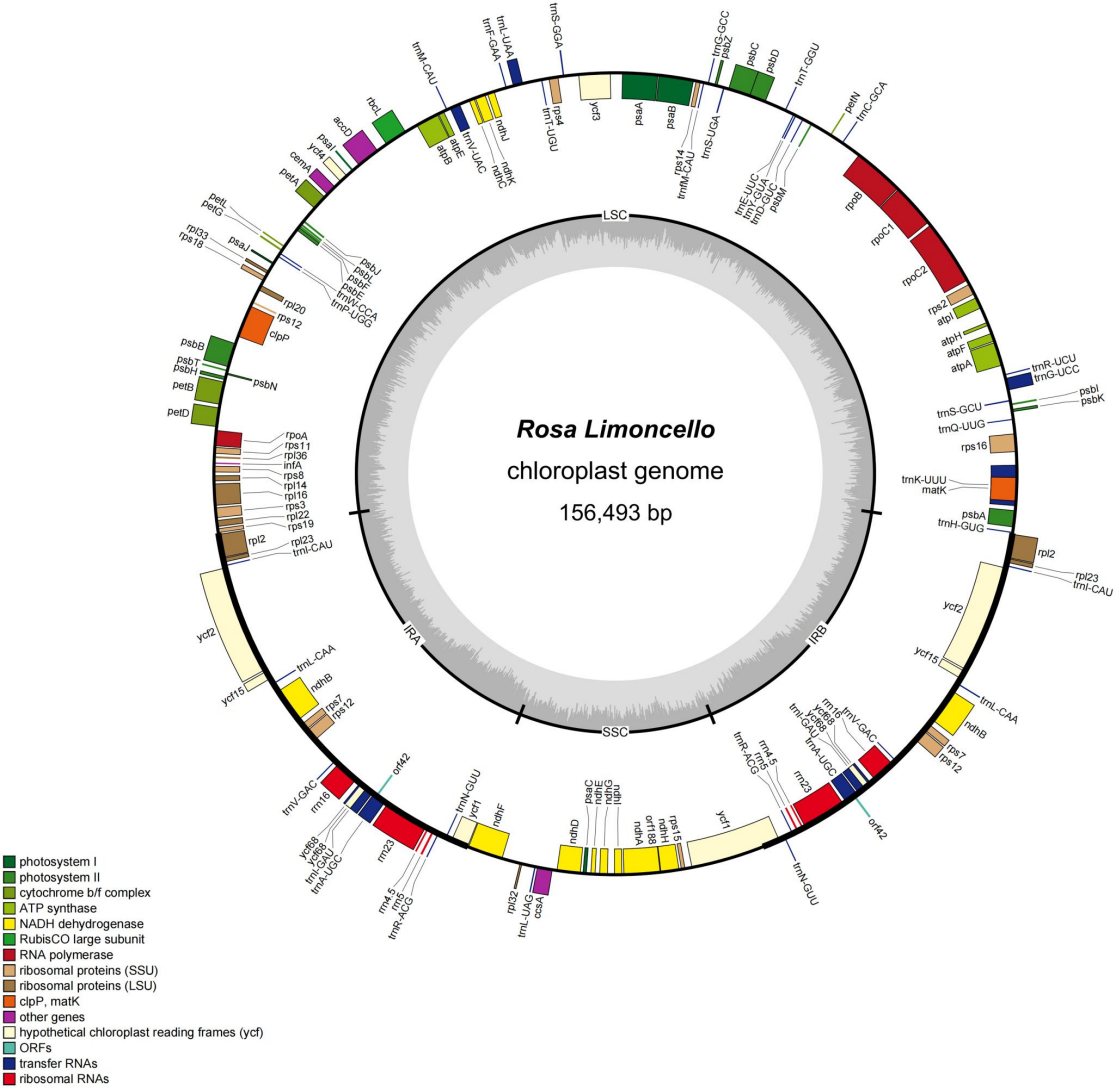
Gene maps of *R*. ‘Limoncello’ genes lying outside the circle are transcribed in a clockwise direction, whereas genes on the inside are transcribed in a counterclockwise direction. Different colors denote known functional groups. The relative GC contents of genomic regions are represented in the inner circle by light gray. LSC, SSC, and IR indicate large single-copies, small single-copies, and inverted repeat regions, respectively.

As the evidence for correct assembly of the genome, the coverage depth figure is provided, and the average depth was 23,857× (Figure S1). The complete chloroplast genome sequence of *R.* ‘Limoncello’ (GenBank: ON000460) was 156,493 bp (Figure 2). It has a circular tetrad structure of the typical angiosperms chloroplast genome. It contained two short inverted repeats (IRa and IRb) regions of 26,052 bp, each separated by a large single-copy (LSC) region of 85,649 bp and a small single-copy (SSC) region of 18,740 bp. The GC content of the IR, SSC, and LSC regions of the genome was 42.72%, 31.33%, and 35.21%, respectively. The GC content of the LSC and SSC regions was lower than that of the two IR regions, which is similar to *Spathiphyllum* ‘Parrish’ (Liu et al. 2019a, 2019b) and *Celosia cristata* (Liu et al. 2020). The chloroplast DNA of *R.* ‘Limoncello’ was predicted to contain 135 genes, including 90 protein-coding genes, eight ribosomal RNA genes, and 37 transfer RNA genes. According to their function, all annotated genes were divided into four main categories: genes related to photosynthesis (*n* = 45), genes related to self-replication (*n* = 74), genes encoding other proteins (*n* = 6), and genes of unknown function (*n* = 10), with 19 of them (ndhB, rpl2, rpl23, rps12, rps7, rrn16, rrn23, rrn4.5, rrn5, trnA-UGC, trnI-CAU, trnI-GAU, trnL-CAA, trnN-GUU, trnR-ACG, trnV-GAC, orf42, ycf2, and ycf68) being double-copy genes. The small exon of *pet*B, *pet*D, and *rpl*16 and the trans-splicing gene *rps*12 have been annotated correctly by CPGView (Liu et al. 2023). Moreover, a schematic map of the cis- and trans-splicing genes was visualized by CPGView (Liu et al. 2023) (Figures S2 and S3).

**Figure 3.**
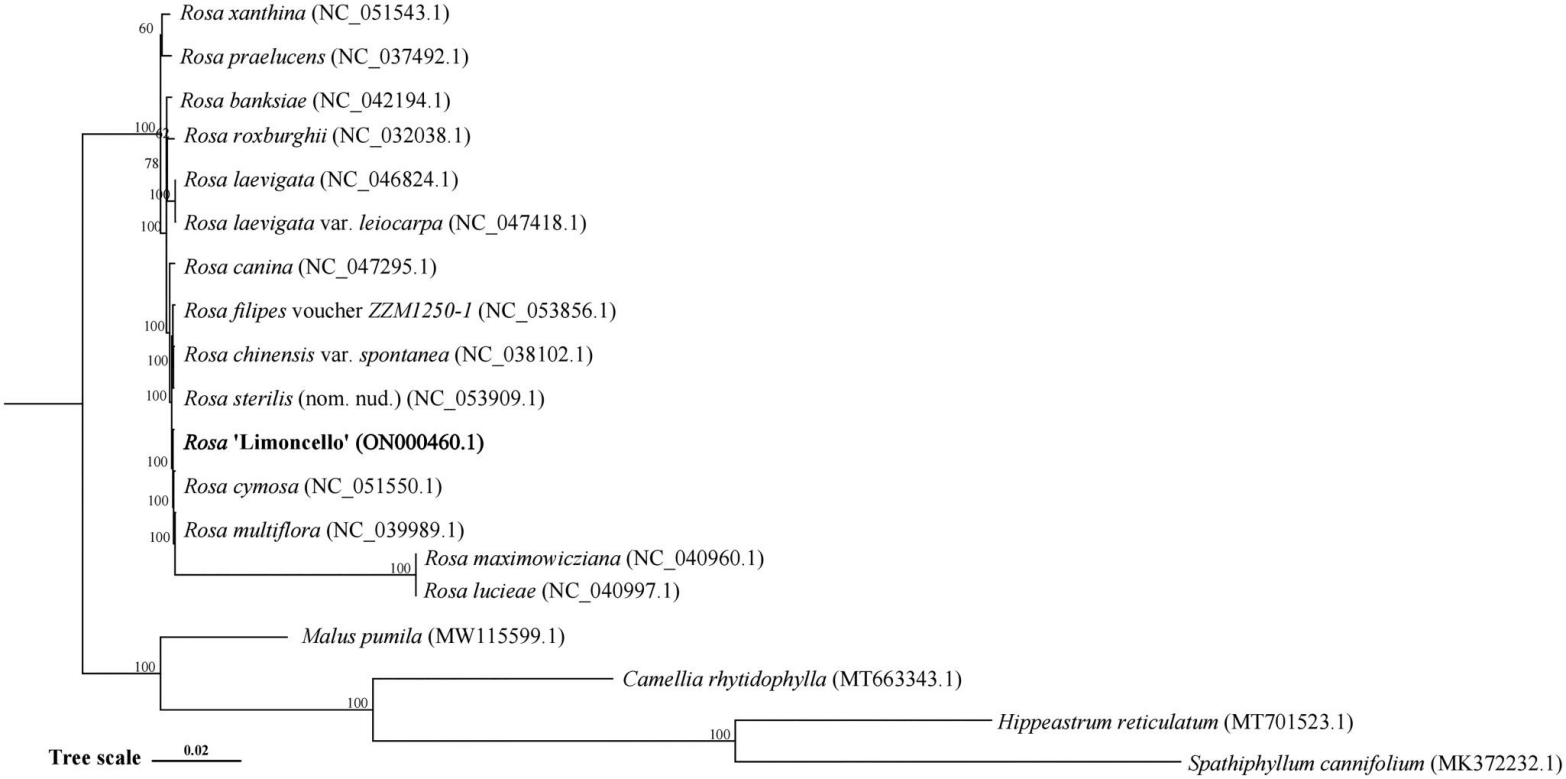
Phylogenetic tree reconstruction of 18 species based on sequences from complete chloroplast genomes. All sequences were downloaded from the NCBI GenBank database. *Spathiphyllum cannifolium* and *Hippeastrum reticulatum* used as outgroups.

To analyze phylogenetic relationships of *Rosa*, we generated a multiple alignment of full-length sequences of chloroplast genomes of *R.* ‘Limoncello’ and 16 other monocots plus two outgroups (*Spathiphyllum cannifolium* and *Hippeastrum reticulatum*) and carried out a ML analysis. *R.* ‘Limoncello’ was found to be closely related to *R. cymosa* (Figure 3).

Information on the complete chloroplast genome sequence of *R.* ‘Limoncello’ obtained in this study can guide phylogenetic studies of the genus *Rosa*.

The phylogenetic tree was constructed using the ML method and bootstrap was performed 1000 times. The number on each branch indicates the boot support value. The following sequences were used: *Rosa xanthina* (NC_051543) (Gao et al. 2020), *R. praelucens* (NC_037492) (Jian et al. 2018), *R. banksiae* (NC_042194) (Wang et al. 2019), *R. roxburghii* (NC_032038) (Wang et al. 2018), *R. laevigata* (NC_046824) (Zhang et al. 2019), *R. laevigata* var.*leiocarpa* (NC_047418) (Sun et al. 2020), *R. canina* (NC_047295) (Yin et al. 2020), *R. filipes* voucher *ZZM1250-1* (NC_053856) (Wang et al. 2020), *R. chinensis* var. *Spontanea* (NC_038102) (Jian et al. 2018), *R. sterilis* (nom.nud.) (NC_053909) (Yan et al. 2021), *R. cymosa* (NC_051550) (Ding et al. 2020), *R. multiflora* (NC_039989) (Zhao and Gao 2020), *R. maximowicziana* (NC_040960) (Jeon and Kim 2019), *R. lucieae* (NC_040997) (Jeon and Kim 2019), *Malus pumila* (MW115599), *Camellia rhytidophylla* (MT663343), *Hippeastrum reticulatum* (MT701523), and *Spathiphyllum cannifolium* (MK372232) (Liu et al. 2019a, 2019b)

## Discussion and conclusions

In this study, we reported and analyzed the first complete chloroplast genome of *R***.** ‘Limoncello’. Our phylogenetic analysis showed that sampled species of the genus *Rosa* formed a monophyletic clade. The results were consistent with previous phylogeny based on plastid markers (Jian et al. 2018). And the chloroplast genome of *R.* ‘Limoncello’ will provide valuable information for further studies and revisions of the genus *Rosa*. Information will be useful for the phylogenetic study of genus *Rosa*, and might also facilitate the genetics and breeding of modern roses.

## Supplementary Material

Supplemental Material

Supplemental Material

Supplemental Material

## Data Availability

Data obtained in this study are available on the NCBI website under accession number ON000460 (https://www.ncbi.nlm.nih.gov/nuccore/ON000460). The associated BioProject, SRA, and Bio-Sample numbers are PRJNA811247, SRR18240725, and SAMN26317739, respectively.
