# Supplementary figures and images for: The complete chloroplast genome sequence of Rosa ‘Limoncello’ (Rosales: Rosaceae)
Source: Mitochondrial DNA B Resour. 2023 Dec 18;8(12):1386–90. doi: 10.1080/23802359.2023.2290854 (PMC10768736; doi:10.1080/23802359.2023.2290854)

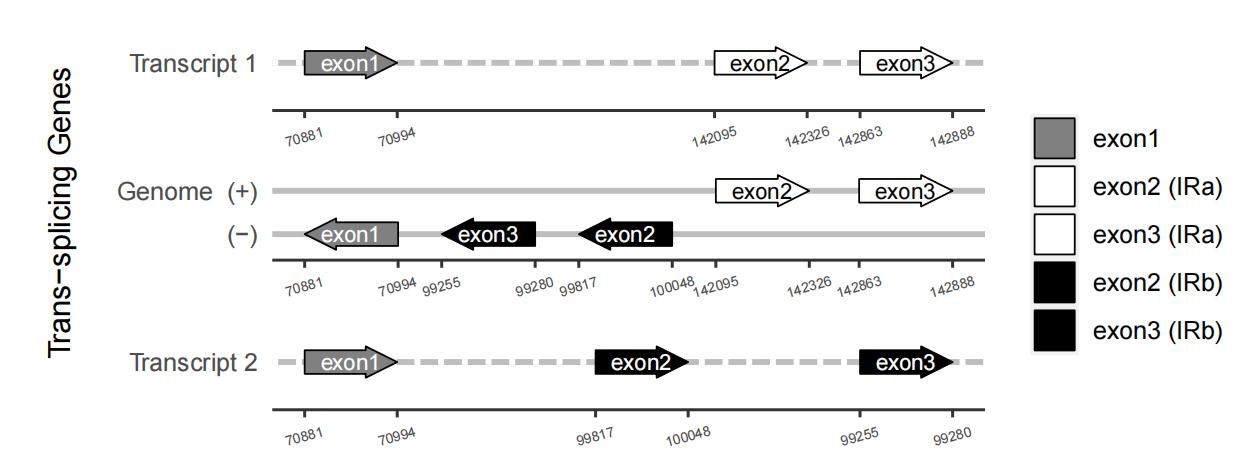

Supplement: Supplemental Material [file TMDN_A_2290854_SM1104.tif]

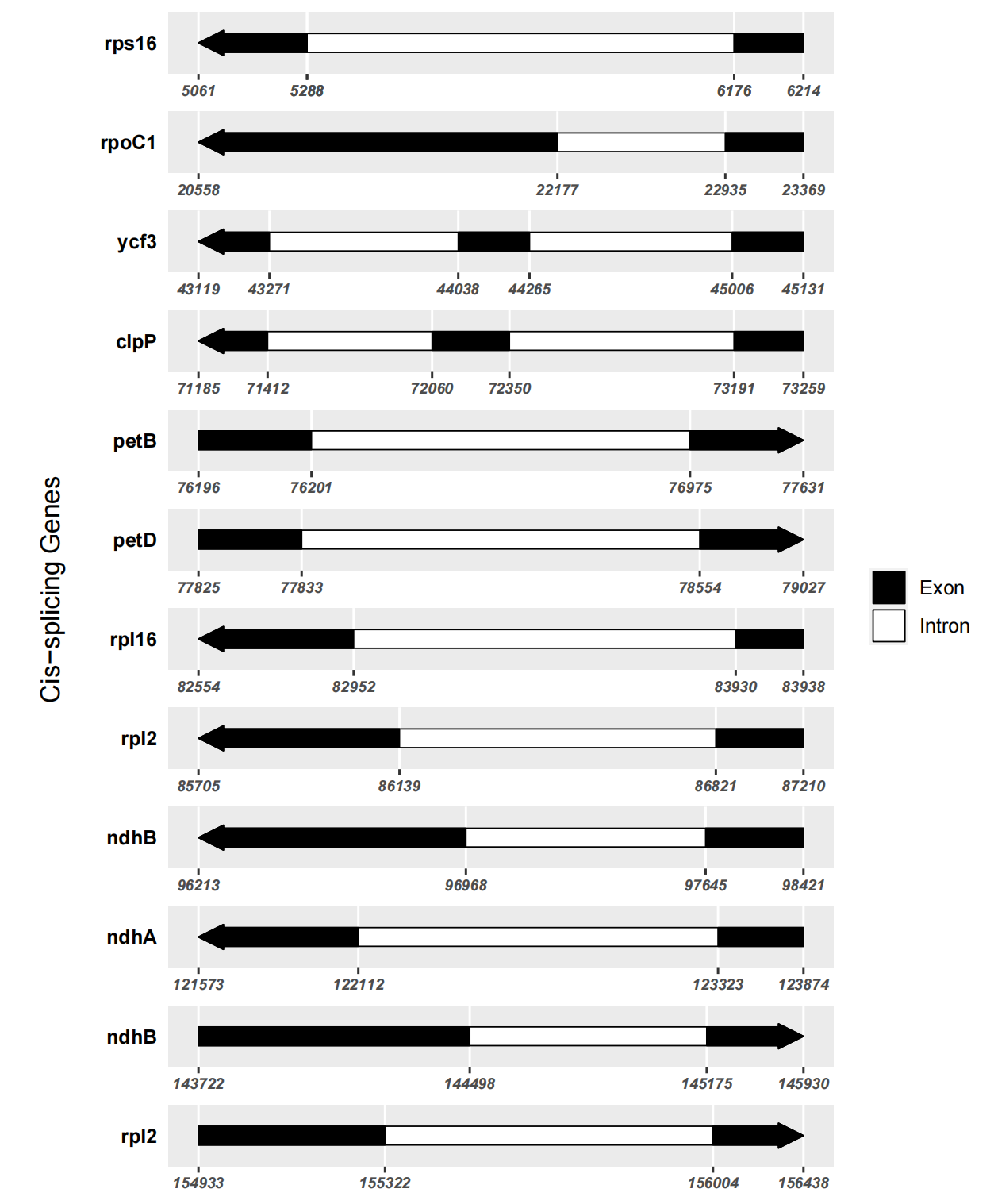

Supplement: Supplemental Material [file TMDN_A_2290854_SM1103.tif]

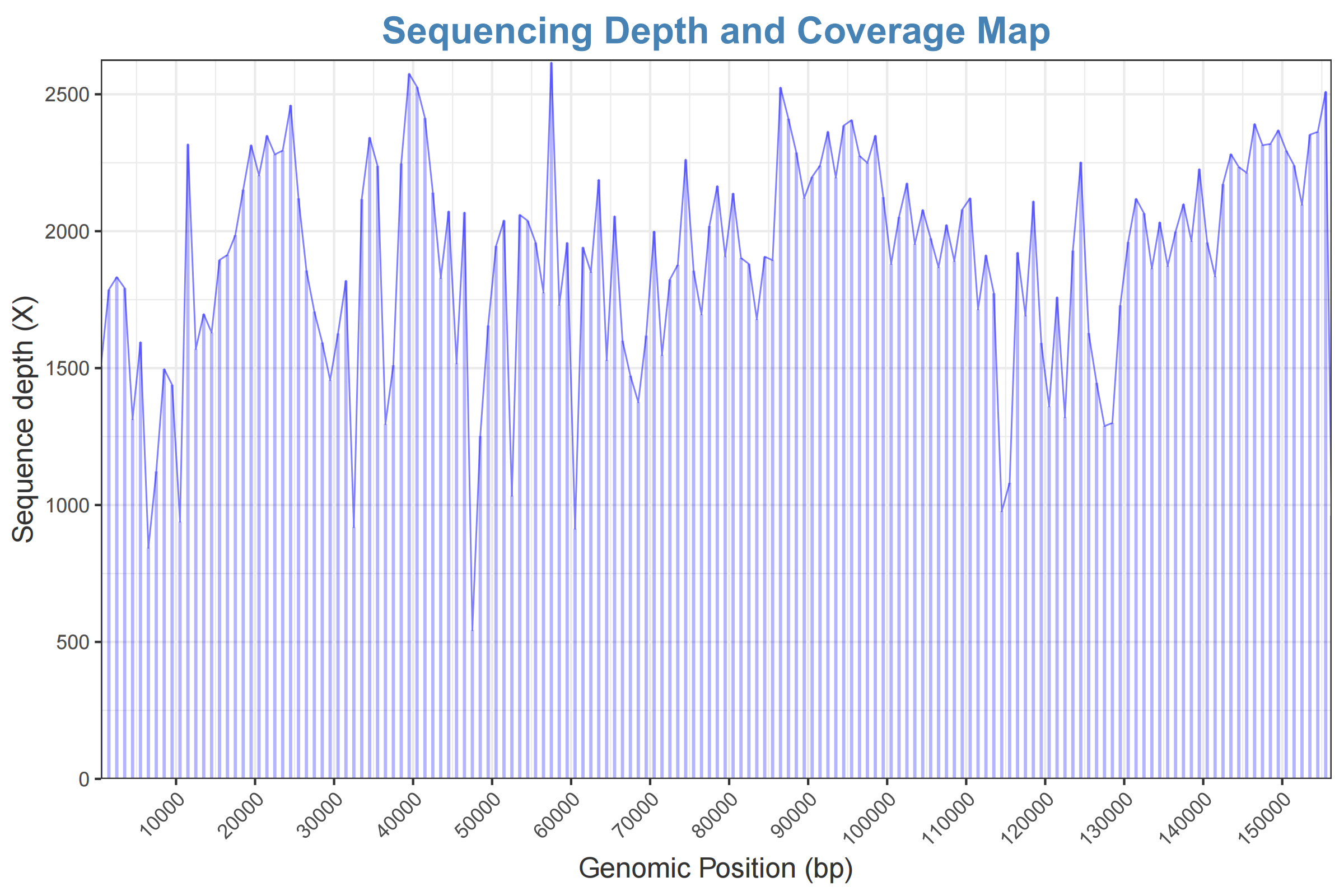

Supplement: Supplemental Material [file TMDN_A_2290854_SM1101.tif]
